# New Miocene Fossils and the History of Penguins in Australia

**DOI:** 10.1371/journal.pone.0153915

**Published:** 2016-04-26

**Authors:** Travis Park, Erich M. G. Fitzgerald, Stephen J. Gallagher, Ellyn Tomkins, Tony Allan

**Affiliations:** 1 School of Biological Sciences, Monash University, Clayton, Victoria, Australia; 2 Palaeontology, Museum Victoria, Melbourne, Victoria, Australia; 3 School of Earth Sciences, University of Melbourne, Victoria, Australia; 4 CSIRO Radiogenic Isotope Facility, North Ryde, Sydney, New South Wales, Australia; Smithsonian Institution, UNITED STATES

## Abstract

Australia has a fossil record of penguins reaching back to the Eocene, yet today is inhabited by just one breeding species, the little penguin *Eudyptula minor*. The description of recently collected penguin fossils from the re-dated upper Miocene Port Campbell Limestone of Portland (Victoria), in addition to reanalysis of previously described material, has allowed the Cenozoic history of penguins in Australia to be placed into a global context for the first time. Australian pre-Quaternary fossil penguins represent stem taxa phylogenetically disparate from each other and *E*. *minor*, implying multiple dispersals and extinctions. Late Eocene penguins from Australia are closest to contemporaneous taxa in Antarctica, New Zealand and South America. Given current material, the Miocene Australian fossil penguin fauna is apparently unique in harbouring ‘giant penguins’ after they went extinct elsewhere; and including stem taxa until at least 6 Ma, by which time crown penguins dominated elsewhere in the southern hemisphere. Separation of Australia from Antarctica during the Palaeogene, and its subsequent drift north, appears to have been a major event in Australian penguin biogeography. Increasing isolation through the Cenozoic may have limited penguin dispersal to Australia from outside the Australasian region, until intensification of the eastwards-flowing Antarctic Circumpolar Current in the mid-Miocene established a potential new dispersal vector to Australia.

## Introduction

Recent studies have enriched our understanding of the evolution and biogeography of penguins (Sphenisciformes) in New Zealand, Antarctica, South America and South Africa [1–8]. Yet Australia remains an inadequately understood region in the global picture of penguin evolution. The Cenozoic history of Australia is unique among southern continents: it has experienced major on-going change in latitudinal position amidst sequential reorganization of ocean circulation patterns resulting in the physical isolation of the continent for most of the last 66 Ma [9]. The history of penguins in Australia is also unusual, having a long (although relatively depauperate) published record spanning the Eocene to Holocene [10,11,12]. Previously described taxa and key specimens include: *Pachydyptes simpsoni* (SAM P14157), from Blanche Point, South Australia (late Eocene) [10,11]; SAM P7158, a left humerus from Witton Bluff, South Australia (late Eocene) [11,12]; SAM P10863, a partial right humerus from Mount Gambier, South Australia (late Oligocene) [11,12]; *Anthropodyptes gilli* (NMV P17167), from near Dartmoor, Victoria (early Miocene) [11,13]; *Pseudaptenodytes macraei* (NMV P26668) from near Minhamite, Victoria (late Miocene) [14,15];? *Pseudaptenodytes minor* (NMV P26669), from Beaumaris, Victoria (late Miocene) [11,14]; and *Tasidyptes hunteri* from Hunter Island, Tasmania (Holocene) [16]. Despite these published specimens, the Australian fossil penguin record lacks substantiated pre-Quaternary evidence of fossil crown taxa contrasting with their occurrence in other regions from the late Miocene onwards [5,7,17–23]. Australia instead only possesses one resident species, the little penguin (*Eudyptula minor*), which has no pre-Quaternary fossil record [24]. The hitherto sparsely sampled late Miocene record of penguins in Australia has hindered the clarification of whether crown spheniscids reached all southern landmasses, and therefore if the global penguin fauna ‘modernized’, by the end of the Neogene.

New material in the form of several penguin humeri from the recalibrated upper Miocene Port Campbell Limestone of Portland, Victoria, Australia has allowed this problem to be explored for the first time. The newly discovered fossils, with emphasis on the nearly complete humerus NMV P221273, are described and compared to other Neogene penguin taxa, and incorporated in a phylogenetic analysis with previously described Australian fossils to address: relationships of Australian fossil penguins to other taxa; when crown group penguins arrived in Australia; and whether Australian fossil penguins follow emerging global trends in penguin faunal evolution.

## Geological Setting

The fossils described here come from a Neogene marine sequence at Portland, Victoria, in the Otway Basin of southeast Australia [25]. There are two fossiliferous units that crop out at Portland: white to light grey carbonate–rich (>96%) Miocene Port Campbell Limestone (PCL), which is unconformably overlain by ferruginized siliciclastic-rich Pliocene Whalers Bluff Formation [26–28]. Both units at Portland have yielded fossil vertebrate assemblages [29]. Isolated bones and teeth from the Pliocene Whalers Bluff Formation represent a moderately diverse vertebrate assemblage of chondrichthyans, cetaceans, pinnipeds, and terrestrial mammals; the fossils are typically orange and brown in colour, as well as often being polished and abraded [29–31]. A diverse assemblage of marine vertebrates has been derived from the underlying PCL along the Portland coastline as well as from submerged exposures in Portland Bay. Vertebrate fossils from the onshore exposures of the PCL typically have finely preserved surface details, with fossils from the more chalky white and grey horizons generally being grey to black in colour; whereas fossils from the more quartz-rich tempestite horizons within the PCL may be yellow and orange in colour. The age and depositional history of the PCL at Portland has until now been inadequately constrained. Hence, a detailed stratigraphic study was carried out in order to refine the age of the PCL strata from which vertebrate fossils were collected.

**Fig 1:**
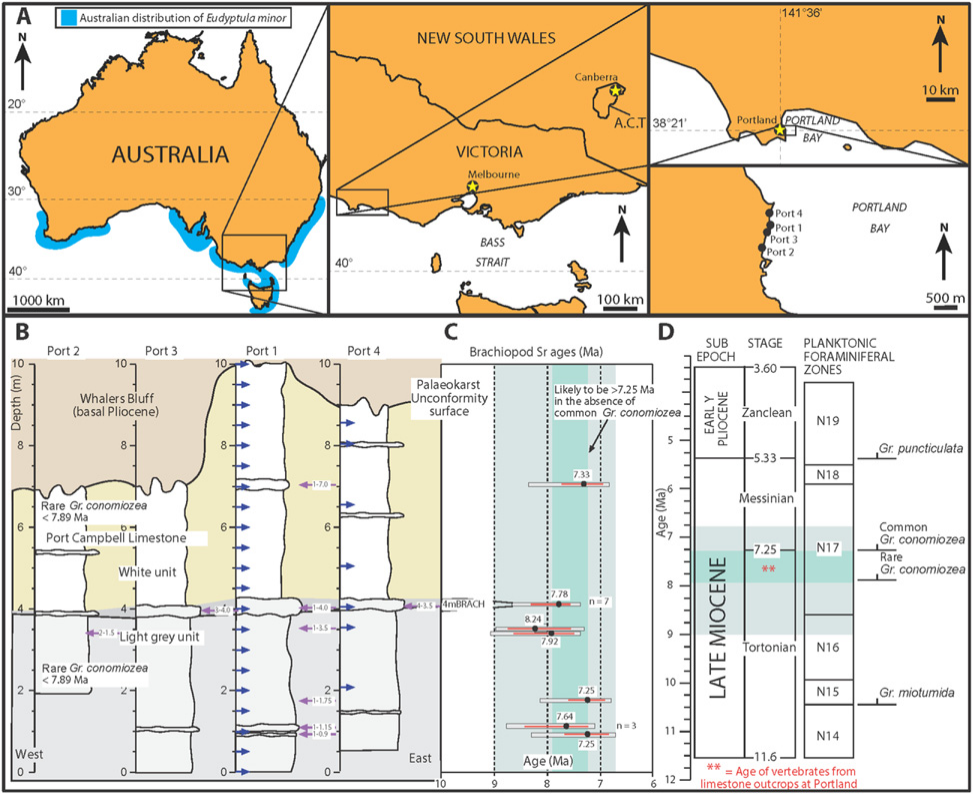
Locality map and stratigraphical section. **(A)** Maps showing the localities of penguin fossils NMV P221273, NMV P232062 and NMV P251637, plus measured stratigraphic sections (Port 1–4) at Portland, Victoria, Australia. **(B)** The stratigraphy of the Portland outcrop. The vertical scale is in metres, the coordinates of each section are: Port 1, 38°20′14″S, 141°36′35″E; Port 2, 38°20′29″S, 141°36′27″E; Port 3, 38°20′18″S, 141°36′32″E; Port 4, 38°20′04″S, 141°36′33″E. The occurrence of *Gr*. *conomiozea* in Port 2 is from [27]. Coarser cross-bedded calcarenite units are indicated. The blue arrows are microfossil samples, the purple arrows are brachiopods samples for Sr analyses. **(C)** Sr ages of brachiopods (see also Tables 1 and 2). Results of replicate analyses of the seven brachiopods from the horizon denoted (4mBRACH) at log level 4 metres are in Table 1. The black circle and red line are the preferred ages with +/- error; the surrounding grey box shows the total age range of each sample. The green shading is the most likely age range of the outcrop; the light blue incorporates the full error of the Sr dates. **(D)** The bio-chronostratigraphy of the Mio-Pliocene [after 35] showing the most likely age of the vertebrate fossils from the Port Campbell Limestone at Portland. Abbreviations: km, kilometres; m, metres; Ma, million years ago; Port, geological section; Sr, strontium.

Four sections were measured and sampled for brachiopods and microfossils (Port 1 to Port 4: Fig 1). Brachiopods were collected for Strontium (Sr) dating. All samples were prepared according to the method outlined in McLaren et al. [32]. They were cleaned and washed using dilute hydrochloric acid. Each brachiopod was powdered using an agate mortar and pestle. The powdered sample was then leached in 3 mL of 10% acetic acid for 1 hour and converted to chloride form. Strontium was separated using ion chromatography. Isotopic analysis was performed using the VG 354 Thermal ionization mass spectrometer at the CSIRO Radiogenic Isotope Facility at North Ryde, Sydney, Australia, with a known reference material NBS987 (^87^Sr/^86^Sr = 0.710235). The results were recalibrated to the standard NBS987 = 0.710248. Results of the isotopic analyses are shown in Tables 1 and 2. Look-up Table Version 4: 08/03 of McArthur et al. [33] was used to obtain the ages of the samples. Sources of age uncertainty include: (i) global ^87^Sr/^86^Sr seawater curve error; (ii) ^87^Sr/^86^Sr measurement error of the reference material; and (iii) the error on the measurement of the ^87^Sr/^86^Sr of the unknown samples. Twenty-eight sediment samples were taken for foraminiferal biostratigraphic analyses. These were processed and analysed using standard micropalaeontological techniques.

**Table 1:** Replicate analyses of two batches of brachiopods from the 4mBRACH and Port 1, P1-1.15 m horizons. The standard error (s.e.) uncertainty is calculated using Student's t test as outlined in [33].

Sample ID (**4mBRACH**)	^87^Sr/^86^Sr	2s error
P1-4a	0.7089290	0.000010
P1-4b	0.7089310	0.000009
P3-4a	0.7089140	0.000009
P3-4b	0.7089210	0.000009
P4-3.5a	0.7089200	0.000007
P4-3.5b	0.7089220	0.000009
P4-3.5c	0.7089170	0.000010
Mean	0.7089220	0.000009
Value of Student's t	2.841	
s.e. at 95% confidence interval	0.0000048	
*n*	*7*	
Ratio normalized to NBS987 = 0.710235	0.7089350	
Sample ID **(Port 1, P1-1.15m)**	^87^Sr/^86^Sr	2s error
P1-1.15a	0.7089200	0.000009
P1-1.15b	0.7089290	0.000009
P1-1.15c	0.7089260	0.000010
Mean	0.7089250	0.000009
Value of Student's t	4.181	
s.e. at 95% confidence interval	0.0000103	
*n*	*3*	
Ratio normalized to NBS987 = 0.710235	0.7089380	

**Table 2:** The age of the brachiopods at Portland.

Sample ID	87Sr/86Sr	2s error	Age range (Ma)	Preferred age (Ma)	*n*
4mBRACH	0.708935	0.0000048	7.37–8.67	7.78+0.52/-0.23	*7*
P1-0.9a	0.708948	0.000011	6.72–8.30	7.25+0.43/-0.51	*1*
P1-1.15	0.708938	0.0000103	7.10–8.78	7.64+0.78/-0.39	*3*
P1-1.75a	0.708948	0.000009	6.79–8.13	7.25+0.35/-0.34	*1*
P1-3.5a	0.708931	0.000009	7.37–9.07	8.24+0.51/-0.69	*1*
P1-7a	0.708946	0.000010	6.83–8.35	7.33+0.40/-0.38	*1*
P2-1.5a	0.708933	0.000009	7.30–8.97	7.92+0.72/-0.44	*1*

Note: measured isotopic ratios normalized to the standard SRM987 = 0.710248. External Precision from measurement of NBS987standard ±0.0020% (±0.000014) (95% confidence limits). Ages calculated from [33]. Preferred ages include the error on the Sr sea-water curve from the Lowess Look-Up Table Version 4: 08/ 03. Age range includes the errors of the look up table and analytical error.

The results of the stratigraphic analyses are shown in Fig 1 and Sr age data are listed in Tables 1 and 2. The outcrop at Portland is dominated by white to light grey limestone with regularly occurring cross-bedded calcarenite units that can be followed along strike for over one kilometre. The lower light grey unit is a bioturbated calcisiltite to calcarenite with minor marl, whereas the upper white unit varies from laminated chalky calcarenite to bioturbated calcisiltite. Invertebrate macrofossils such as irregular echinoderms and brachiopods are scattered throughout the section and are concentrated in the cross-bedded calcarenite units. Biostratigraphic analyses reveal minor planktic foraminifera such as *Globorotalia miotumida*. This taxon typifies the late Miocene strata of southeast Australia [34] where it first occurs after 10.5 Ma. Rare *Globorotalia conomiozea* has been reported from the top and base of Port 2 by Mallett [27]; the present study was unable to repeat this result. Nevertheless, Mallett’s [27] results suggest that this part of the section may be younger than 7.89 Ma [35]. The rarity of *Gr*. *conomiozea* in the section may be related to the shallow water facies (this taxon is a deep-dwelling species [36]). Yet it is also likely that this section is older than the first common appearance of this taxon at 7.25 Ma [35]. The spread of Sr ages for the section ranges from a minimum of 6.72 to a maximum of 9.07 Ma (the light blue envelope on Fig 1C and 1D). However, there is a strong clustering of ages between 7.25 and 7.92 Ma. This overlaps with the microfossil ages and suggests most of the outcropping Portland limestone is in this age range, and therefore was deposited in the late Tortonian.

## Methods

### Institutional Abbreviations

NMV B, NMV R, NMV W, Museum Victoria Ornithology Collection, Melbourne, Australia; NMV P, Museum Victoria Palaeontology Collection, Melbourne, Australia; SAM P, South Australian Museum Palaeontology Collection, Adelaide, Australia.

Osteological terminology and terms of orientation follow Baumel and Witmer [37]. NMV P221273 was prepared by EMGF. Measurements were taken by TP using Mitutoyo Absolute Digimatic CD-600CSX callipers. For photography, fossil specimens were coated in a sublimate of ammonium chloride (NH_4_Cl) by the authors. Photographs were taken using a Nikon D90 digital SLR camera with a Nikon Nikkor 60 mm micro lens by TP and EMGF. In order to identify NMV P221273, it was compared with the humeri of 36 post-Oligocene taxa. No taxa older than the Miocene were compared as they were considered to be too anatomically dissimilar as to make any meaningful comparisons. Twelve of the 19 extant species used for comparative analysis were obtained from the Museum Victoria Ornithology collection; the character states for the remaining seven extant species were taken from the data matrix of Ksepka et al. [2]. As unnamed specimens constitute some of the ‘taxa’ included in the phylogenetic analysis the term operational taxonomic unit (OTU) is used in the phylogenetic analysis section. Photographs of previously described Australian fossil penguins were published by Park and Fitzgerald [11]. Comparisons with fossil taxa and character codings were made using specimens from the Museum Victoria Palaeontology Collection and images and text from relevant published literature.

### Comparative Material

NMV B30374, *Aptenodytes patagonicus*; NMV B18320, *Aptenodytes forsteri*; NMV B7885, *Eudyptes chrysocome*; NMV W6357, *Eudyptes moisleyi*; NMV B7889, NMV B7879, *Eudyptes pachyrhynchus*; NMV B19894, *Eudyptes robustus*; NMV B30164, NMV B6232, *Eudyptes schlegeli*; NMV W8850, *Eudyptula minor*; NMV R6546, *Pygoscelis adeliae*; NMV B12925, *Pygoscelis antarctica*; NMV B7877, *Pygoscelis papua*; NMV B18433, *Spheniscus demersus*; NMV P17167, *Anthropodyptes gilli*.

## Results

### Systematic Palaeontology

Aves Linnaeus, 1758

Sphenisciformes Sharpe, 1891

Sphenisciformes gen. et sp. indet.

### Referred Material

**Fig 2:**
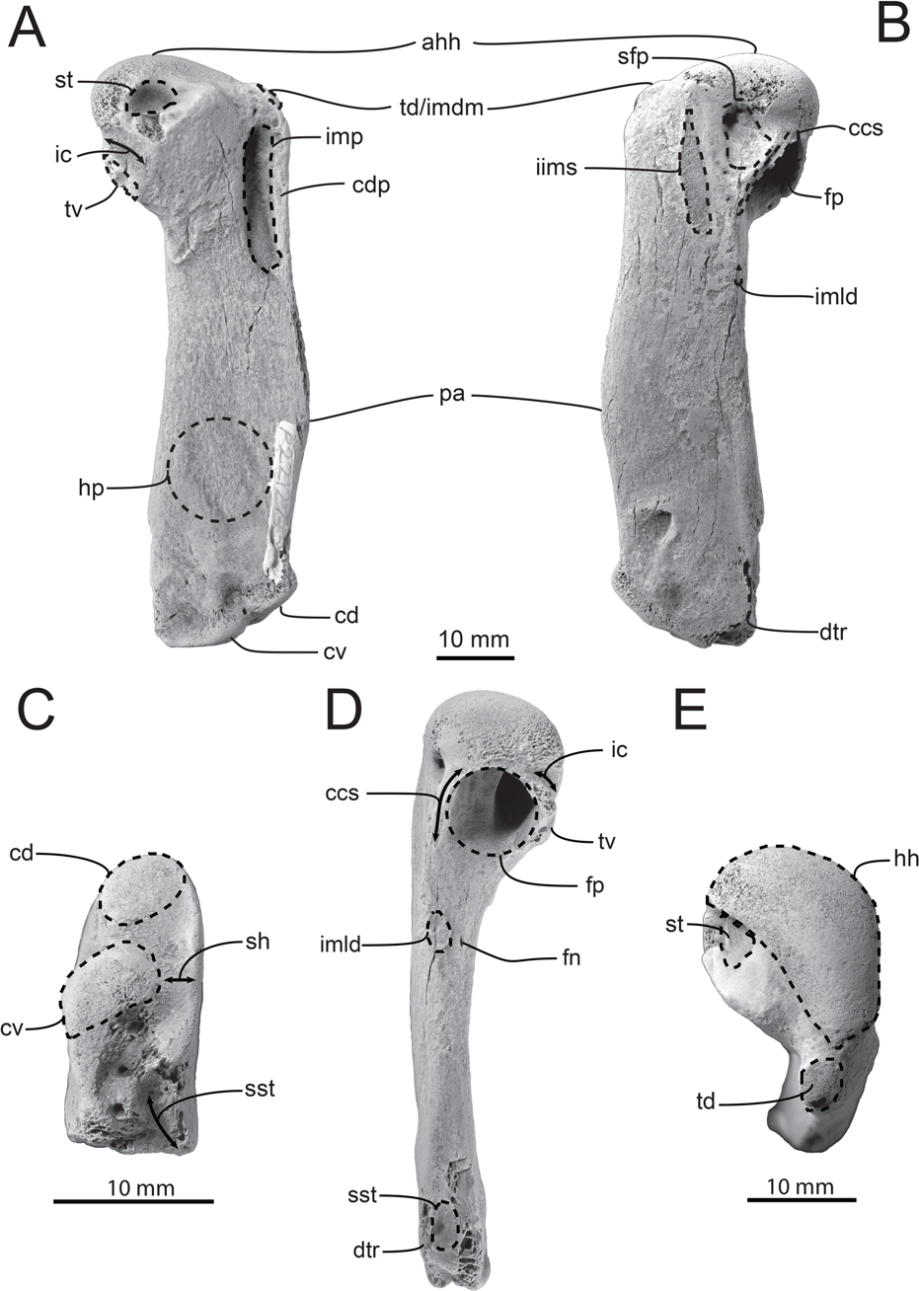
NMV P221273, left humerus. A, Ventral; B, dorsal; C, distal, D, caudal; and E, proximal views. Abbreviations: ahh, apex of humeral head; ccs, coracobrachialis caudalis scar; cd, condylus dorsalis; cv, condylus ventralis; dtr, dorsal trochlear ridge; fp, fossa pneumotricipitalis; hh, humeral head; hp, humeral plexus; ic, incisura capitis; iims, impressio insertii musculi supracoracoideus; imdm, impressio musculi deltoideus minor; imld, impressio musculi latissimus dorsi; imp, impressio musculi pectoralis; pa, preaxial angle; sfp, secondary fossa pneumotricipitalis; sh, shelf adjacent to condylus ventralis; sst, sulcus scapulotricipitalis; st, sulcus transversus; td, tuberculum dorsale; tv, tuberculum ventrale. Scale bars represent 10 mm. Specimen whitened with ammonium chloride.

**Fig 3:**
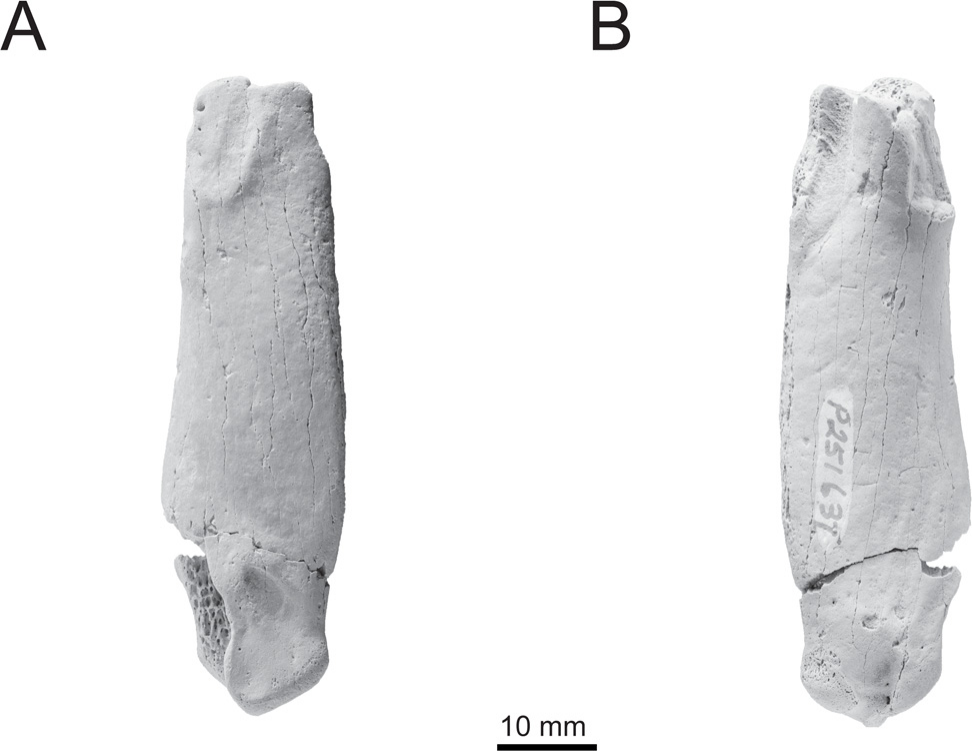
Referred material. NMV P251637, partial right humerus in: A, dorsal; and B, ventral views.

NMV P221273, left humerus, collected by S. Wright in June 2006 (Fig 2); NMV P251637, partial right humerus, collected by D. Aitken and T. O’Keefe (Fig 3).

### Locality

Beach on western side of Portland Bay, at Portland, Victoria; 38°20’28”S, 141°36’30”E (Fig 1).

### Horizon and Age

Port Campbell Limestone; late Miocene (Tortonian: 7.25–7.92 Ma) (Fig 1). NMV P221273 is from the lower light grey unit of the Port Campbell Limestone at Portland, whereas NMV P251637 is from the upper white unit of the same formation.

### Diagnosis

A left humerus with the following combination of character states unique to the Sphenisciformes: head developed proximally and reniform in proximal view; fossa pneumotricipitalis deep without pneumatic foramen; impressio insertii m. supracoracoideus elongated with long axis subparallel to long axis of shaft; demarcation of sulcus scapulotricipitalis a trochlear ridge for articulation with os sesamoideum m. scapulotricipitalis. The flattened shape of the humerus also excludes it from virtually all avian groups, except for the Plotopteridae. Differences between NMV P221273 and other taxa are as follows, with character states of the respective taxa given in brackets. Differs from *Palaeospheniscus* and *Eretiscus tonnii* in: condylus ventralis flattened (projecting and rounded); differs from *Marplesornis novaezealandiae* in absolute size (~10% larger), pit for ligament insertion on proximal surface adjacent to head is absent (deep); differs from all extant genera in having a moderate gap between impressio insertii m. supracoracoideus and insertii m. latissimus dorsi (small gap or confluent). Differs from *Pseudaptenodytes macraei* in absolute size (~20% larger), having a distinct preaxial angle (margo cranialis is curved), a rounded ventral portion of the fossa pneumotricipitalis (elliptical ventral portion of the fossa pneumotricipitalis).

### Description

NMV P221273 is a near complete left humerus (Fig 2), lacking only the trochlear ridges and the edge of the proximal border of the fossa pneumotricipitalis. Significant dimensions are in Table 3. In proximal view (Fig 2E), the head is well developed proximally and reniform in shape. The tuberculum dorsale is oval in outline and projects slightly proximally in dorsal view. The pit for ligament insertion adjacent to the humeral head is absent [38]; this feature is present in every extant species except *Pygoscelis antarctica* and *P*. *adeliae* (it is variably developed in *Aptenodytes patagonicus*) [2].

**Table 3:** Measurements of NMV P221273.

Measurement	Value
total length	80.80
craniocaudal width at level of distal edge of tricipital fossa	16.54
craniocaudal width at level of preaxial angle	20.40
craniocaudal width at level of proximal edge of radial condyle	19.64
dorsoventral thickness at level of tricipital fossa	17.44
dorsoventral thickness at level of preaxial angle	7.40
dorsoventral thickness at level of proximal edge of radial condyle	8.73
trochlear angle	50°
preaxial angle	12°

In ventral view (Fig 2A), the sulcus transversus is distal to the articular surface of the head and its shelf runs almost perpendicular to the long axis of the shaft of the humerus. The distal border of the sulcus transversus is slightly damaged, but it is clearly separated from the incisura capitis by a thin ridge of bone. The impressio m. pectoralis is a deep oblong fossa and the crista deltopectoralis is well defined and projects ventrally. The shaft itself is not sigmoid (unlike some late Eocene taxa [39]) and increases in craniocaudal width distally, becoming widest at the preaxial angle (Table 3). The preaxial angle is distinct although less pronounced than that of *Aptenodytes forsteri*. The presence of the humeral plexus [40] is faint but visible on the ventral surface of the shaft as three indistinct sulci.

Dorsally (Fig 2B), the apex (the most proximal point) of the humeral head is situated caudally rather than closer to the midline as in more basal stem species. The insertii m. supracoracoideus is an elongate, shallow, oblong facet that runs slightly obliquely to the long axis of the shaft of the humerus. It is separated from the almost completely eroded insertii m. latissimus dorsi insertion by a moderate gap of 5.16 mm. This feature is clearly apparent in NMV P251637 (Fig 3). The incisura capitis does not extend onto the secondary fossa pneumotricipitalis, which possesses a small but moderately deep pit, the presence of which appears to be variable in other species. The coracobrachialis caudalis scar is flat and elongate. It is oriented obliquely and contacts the distal margin of the head.

Viewed caudally (Fig 2D), the tuberculum ventrale is oriented ventrocaudally. The proximal border of the fossa pneumotricipitalis is concave, although the outermost margins of the border are eroded so degree of projection cannot be determined. The fossa pneumotricipitalis itself is strongly bipartite, with the dorsal fossa having a larger volume than the ventral fossa. Whilst the fossa pneumotricipitalis is deeply excavated, it is less so than extant taxa. The border of the ventral fossa is positioned more caudally than in extant taxa, with the dorsal fossa forming a flattened surface dorsal to it, a morphology also found in *Marplesornis novaezealandiae* (however this feature is difficult to ascertain for other taxa from published figures, therefore limiting its phylogenetic implications at present). The foramen nutricum is located on the caudal surface of the shaft, 13.43 mm distal to the distal border of the fossa pneumotricipitalis.

At the distal end of the shaft (Fig 2D), the middle and ventral trochlear ridges have been eroded. The bases of the ridges remain however and demarcate the position of the sulcus scapulotricipitalis. The dorsal trochlear ridge is the least eroded and enough of it is preserved to determine that it would not have extended past the caudal margin of the shaft, similar to *Spheniscus*. The angle between the long axis of the shaft and the tangent of the condylus dorsalis and condylus ventralis is 50°, all extant Sphenisciformes have an angle >45° [39]. The condylus ventralis is flattened and has a much greater dorsoventral width than the adjacent shelf, resulting in a high (>1.3) condyle width to shelf width ratio.

### Comparisons

NMV P221273 shares the following synapomorphies with all taxa more crownward than *Platydyptes*: a ventrally positioned apex of the humeral head; a clear separation of the incisura capitis and the sulcus transversus; a ventrocaudally oriented tuberculum ventrale; a bipartite fossa tricipitalis; a flattened condylus ventralis; and a ratio of condyle width: shelf width >1.3. NMV P221273 is considered conspecific with NMV P251637, being identical in size and sharing an impressio insertii m. supracoracoideus and insertii m. latissimus dorsi separated by a moderate gap.

NMV P221273 differs from *A*. *gilli*, and all crown taxa by having the impressio insertii m. supracoracoideus and insertii m. latissimus dorsi separated by a moderate gap. NMV P221273 differs from *A*. *gilli*, *Inguza predemersus*, *E*. *tonnii*, *Pa*. *patagonicus*, *Pa*. *bergi*, *M*. *mirandus*, *Tereingaornis moisleyi*, *Aptenodytes*, *Pygoscelis*, and *Eudyptes* by having a dorsal trochlear ridge that does not extend beyond the humeral shaft (*Eudyptula minor*, *S*. *demersus* and *S*. *mendiculus* vary for this [41]). NMV P221273 differs from *Dege hendeyi*, *I*. *predemersus*, *A*. *forsteri*, *Py*. *papua*, *Megadyptes antipodes*, *Eudyptes*, *E*. *minor* and *Spheniscus* by lacking a pit for ligament insertion adjacent to the humeral head (*A*. *patagonicus* is variable for this character). NMV P221273 differs from *Spheniscus* by having a concave proximal margin of the fossa pneumotricipitalis (*E*. *minor* varies for this character). NMV P221273 differs from *A*. *gilli*, *E*. *tonnii* and *Pa*. *patagonicus* by having a condylus ventralis that is flattened. NMV P221273 differs from *A*. *gilli* and *Pa*. *antarcticus* by having a bipartite fossa pneumotricipitalis. NMV P221273 differs from *A*. *gilli* by having a ratio of the shelf width adjacent to the condylus ventralis and the condylus ventralis > 1.3, a shaft that widens distally and by having an angle between the long axis of the humeral shaft and the tangent of condylus dorsalis and condylus ventralis greater than 45°. NMV P221273 differs from *I*. *predemersus* by having a preaxial angle that is distinct. NMV P221273 differs from *Ps*. *macraei* by possessing a more rounded ventral portion of the fossa pneumotricipitalis and a margo cranialis with a distinct preaxial angle [11]. NMV P221273 differs from? *Pseudaptenodytes minor* by having a more robust shaft, a more distinct preaxial angle and is ~10% smaller in absolute size. NMV P221273 differs from *Pa*. *antarcticus* by having the apex of the humeral head located caudal to the midline of the shaft and a tuberculum ventrale that is oriented ventrocaudally. NMV P221273 is most similar to *Palaeospheniscus*, *E*. *tonnii*, *M*. *novaezealandiae* and crown group taxa by having: a ventrally positioned apex of the humeral head; a clear separation of the incisura capitis and the sulcus transversus; a ventrocaudally oriented tuberculum ventrale; a bipartite fossa tricipitalis; and a ratio of condyle width: shelf width >1.3. NMV P221273 cannot be conclusively allied to any described genus based on morphological comparisons alone due to conflicting character combinations and the limited material at hand. NMV P221273 is thus referred to Sphenisciformes gen. et. sp. indet.

## Phylogenetic Systematics

This phylogenetic analysis aims to resolve: (1) the relationships of Australian fossil penguins to each other and other taxa; (2) the timing of the arrival of crown group penguins in Australia; and (3) identify any biogeographic implications of Australian fossil penguin relationships, specifically whether the Australian penguin fauna is the result of a single dispersal event to Australia or represents independent dispersals throughout the Cenozoic.

### Analysis protocol

**Fig 4:**
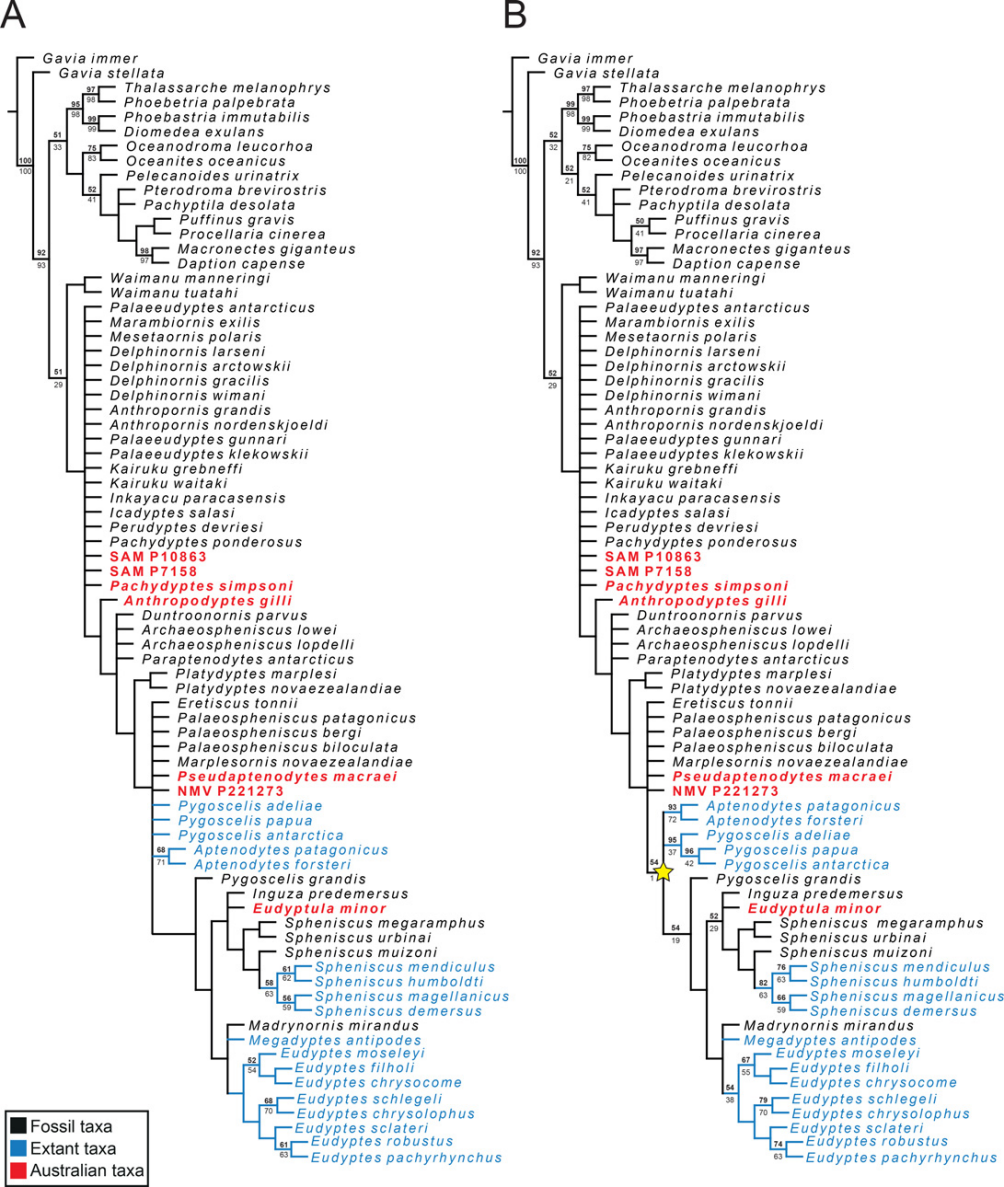
Phylogeny of Sphenisciformes including Australian OTUs. Strict consensus of: A, 194 most parsimonious trees with an unconstrained search; and B, 219 most parsimonious trees with crown Sphenisciformes constrained (denoted by the yellow star) in a constrained search. Standard bootstrap (1000 replicates) absolute frequency values are in bold above their respective nodes and symmetric resampling (1000 replicates) frequency difference values are in regular font below their respective nodes. Best score reached was 5377 for both phylogenies.

The total number of operational taxonomic units (OTUs) included in the data matrix is 76: 61 penguin taxa (all 19 extant species, 39 fossil species and three unnamed specimens) and 15 outgroup taxa (two Gaviiformes and 13 Procellariiformes). In all analyses, the trees were rooted to the Gaviiformes following the procedure of Ksepka et al. [2]. This study analyses 245 morphological characters (26 pertaining to the humerus directly) plus 8145 DNA characters from the 12S, 16S, COI, cytochrome b and RAG-1 genes: a total matrix of 8390 characters, adapted from Ksepka et al. [2]. Their matrix was not modified apart from the addition of the Australian fossil taxa. Phylogenetic analyses were performed using TNT 1.1 (no taxon limit) [42], using a new technology search strategy using 10 000 additional replicates with sectorial and tree fusing options checked. Characters were equally weighted and any zero-length branches were collapsed. The consistency and retention indices were applied to the strict consensus tree in order to determine the degree of homoplasy (analogous traits) and amount of character state similarity across taxa which is interpretable as synapomorphy respectively [43]. Both standard bootstrapping and symmetric resampling (33%) analyses were run for 1000 replicates each, with the values being represented by absolute frequencies and frequency differences respectively. The initial analysis found that the inclusion of *Pseudaptenodytes macraei* resulted in the paraphyly of extant *Pygoscelis*, a clade that is otherwise well supported by numerous molecular [44,45] and morphological [23, 39,46,47] analyses. We therefore ran a constrained analysis with the crown clade constrained using the same parameters as above to resolve this issue. Both phylogenies are shown in Fig 4. The following results and discussion are based on the phylogeny obtained in the constrained analysis (Fig 4B).The phylogenetic matrix file can be found in the supporting information (S1 File).

### Results

The parsimony analysis resulted in 194 (unconstrained) and 219 (constrained) most parsimonious trees (MPTs) with a best score of 5377; consistency index (CI) of 0.536; and a retention index (RI) of 0.698. The CI and RI values were the same in both analyses. Specific results pertaining to Australian OTUs are discussed below.

*Pachydyptes simpsoni*: In our analysis *Pachydyptes simpsoni* is resolved in a polytomy (Fig 4) with *Marambiornis exilis*, *Mesetaornis polaris*, *Delphinornis* spp., SAM P7158, SAM P10863, *Anthropornis* spp., *Palaeeudyptes* spp., *Inkayacu paracasensis*, *Icadyptes salasi*, *Perudyptes devriesi*, *Pachydyptes ponderosus* and *Kairuku* spp. This position does not reveal a most parsimonious ancestral area for *P*. *simpsoni*. However, a relationship between *P*. *simpsoni* and the Seymour Island taxa has been hypothesised by Acosta Hospitaleche et al. [48], wherein stem taxa dispersed from the Weddellian Province [49] to Australia (and South America) when there was a shallow, warm ocean current (the “Weddellian Current” of Acosta Hospitaleche et al. [48]) during the early Eocene. Furthermore, reanalysis of Antarctic material [4] has shown that the late Eocene *Anthropornis* also possessed the same derived morphology of the carpometacarpus. This is significant as *P*. *simpsoni* was referred to *Anthropornis nordenskjoeldi* by Jenkins [50] and could be interpreted as supporting the contention that *P*. *simpsoni* is a member of the genus *Anthropornis*. This may be an artefact of the poorly preserved holotype material of *P*. *simpsoni*, or perhaps *P*. *simpsoni* is not a species of *Anthropornis*, with the shared derived morphology of the carpometacarpus being convergent. Furthermore, the preserved material of the *P*. *simpsoni* holotype contains elements that are poorly known in other late Eocene taxa (e.g. radius, carpometacarpus), which hinders comparisons and clarification of relationships.

SAM P7158 (cf. *Palaeeudyptes*): SAM P7158 was initially designated as *Palaeeudyptes* cf. *antarcticus* in previous studies [51,52]. More recent work has classified SAM P7158 as cf. *Palaeeudyptes* [53] based on comparable Antarctic taxa given that *P*. *antarcticus* is known only from a tarsometatarsus [54] and therefore is not comparable to other elements. SAM P7158 occupies same polytomy as *P*. *simpsoni*.

SAM P10863 (Sphenisciformes gen. et. sp. indet.): SAM P10863 was initially referred to Spheniscidae gen. et. sp. indet. by Simpson [12]. SAM P10863 differs from *Pachydyptes* and *Icadyptes* by having a shaft that is more slender. SAM P10863 differs from *Anthropornis* by having a shaft that is less sigmoid in dorsal view. SAM P10863 differs from *Palaeeudyptes klekowskii* by having a humeral head that is relatively larger and more rhomboid. SAM P10863 differs from *Anthropodyptes gilli* by having a smaller fossa pneumotricipitalis SAM P10863 differs from *Kairuku* by having a more distally extended impressio insertii m. supracoracoideus, more curved cranial edge of the humeral shaft and different angle of the sulcus formed from the contiguous sulcus ligamentum transversus and incisura capitis. SAM P10863 is smaller in length than *K*. *grebneffi* but is almost identical in size to *K*. *waitaki*. SAM P10863 is designated here as Sphenisciformes gen et. sp. indet.

*Anthropodyptes gilli*: *Anthropodyptes gilli* is positioned one node basal to a polytomy consisting of *Duntroonornis parvus*, *Archaeospheniscus* spp. and *Paraptenodytes antarcticus*. In terms of humeral morphology, *A*. *gilli* is by far the most archaic known taxon from the Miocene. It is similar in morphology to *Kairuku*; differing mainly in the lack of the elongate depression near the caudal margin of the ventral face of the shaft (164: 0) and also the oblique angle of the cranial margin proximal to the preaxial angle. Other early Miocene taxa outside Australia are more derived morphologically than *A*. *gilli*, possessing a larger angle between the main axis of the humeral shaft and the tangent of the ulnar and radial condyles. *Anthropodyptes gilli*, on the other hand, retains the plesiomorphic humeral morphology typical of Paleogene taxa.

NMV P221273 (Sphenisciformes gen. et. sp. indet).: NMV P221273 is placed in a polytomy with *Palaeospheniscus* spp., *Eretiscus tonnii*, and *Pseudaptenodytes macraei* one node basal to crown Sphenisciformes. In terms of humeral morphology, only one unambiguous morphological character supports exclusion of this cluster of taxa from crown Sphenisciformes (158: 1, impressio insertii m. supracoracoideus and insertii m. latissimus dorsi separated by a moderate gap). Another ambiguous character (150: 0, lacking a pit for ligament insertion adjacent to the humeral head (*Aptenodytes patagonicus* is variable in this character)) also supports this position. As noted above, NMV P221273 shares with *M*. *novaezealandiae* a similar morphology of the fossa pneumotricipitalis, hinting at an affinity. The lack of comparative figures showing this feature in the literature prevents further investigation at present.

*Pseudaptenodytes macraei*: This is a diagnosable taxon possessing at least two clear autapomorphies: a flattened elliptical ventral portion of the fossa tricipitalis and a curved margo cranialis lacking a preaxial angle [15]. As noted above, *P*. *macraei* is placed one node basal to crown Sphenisciformes in the phylogenetic analysis. Based on its morphological features it is unlikely to be a crown spheniscid, with the caveat that more complete referred specimens are needed to more accurately resolve the relationships of this unusual species.

Crown Sphenisciformes: This analysis recovers one Australian OTU as a crown group penguin: the living species *Eudyptula minor*. Its phylogenetic position is generally consistent with previous studies [2,39].

One other named Australian crown sphenisciform, *Tasidyptes hunteri* was not included in the analysis. This subfossil species, dated to 760 ± 70 ybp [16], is comprised of an adult synsacrum (holotype), a tarsometatarsus (paratype), a juvenile synsacrum and a coracoid (referred material). These elements are from different ontogenetic stages and were found in multiple stratigraphic layers of an aboriginal midden, meaning that the paratype and referred material are not strictly comparable with the holotype. *T*. *hunteri* differs from *Eudyptula* and *Megadyptes* by having: a caudal part of the synsacrum with relatively broader fused vertebrae and long slender lateral processes; and the lateral foramen vasculare proximale situated more distal than the medial foramen vasculare proximale on the plantar surface of the tarsometatarsus [16]. Furthermore, the coracoid and tarsometatarsus are indistinguishable from *Eudyptes*, although the long slender lateral processes of the holotype synsacrum may be a diagnostic character [53]. We therefore recommend restricting the hypodigm of this taxon to the holotype synsacrum only.

## Discussion

Examination and analysis of new and existing Australian fossil penguin material has provides new insights into their evolutionary history. Australian fossil specimens are widely distributed in this phylogeny; suggesting multiple dispersals of penguins to Australia during the Cenozoic, a biogeographic pattern mirrored by the fossil record of penguins in South Africa [7]. However, the late survival of giant penguins and inferred recent arrival of crown group penguins suggest that the Australian penguin fauna has followed an evolutionary trajectory different from other regions. The disparate positions of Australian fossil penguins in the phylogeny and their relationship to global trends in penguin evolution and the physical evolution of Australia continent are discussed below.

### Biogeography of Australian penguins

The scattered positions of Australian penguin taxa across the phylogeny suggests that rather than a single dispersal to Australia followed by an endemic, there have instead been multiple independent dispersals to Australia throughout the course of the Cenozoic. There is no evidence suggesting that any of the Australian taxa are more closely related to each other than to other taxa, although more complete remains of these taxa may reveal a sister group relationship between some species (e.g., *Pseudaptenodytes macraei* and NMV P221273).

### The modernisation of the global penguin fauna

**Fig 5:**
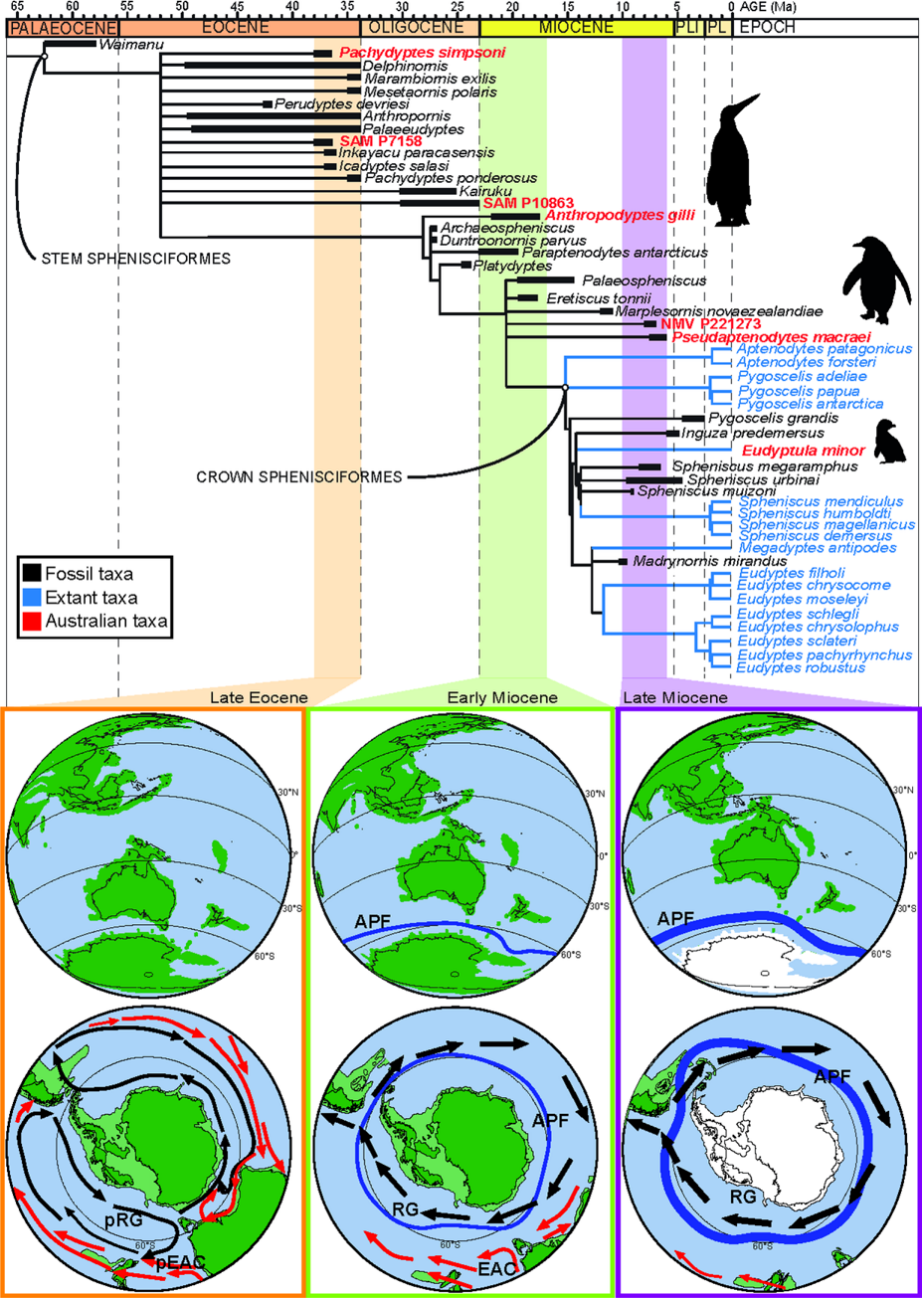
Synthesis of Australian penguin evolution. Stratigraphically-calibrated phylogeny of Sphenisciformes correlated with tectonic movements and changing ocean circulation in the southern hemisphere showing how: (1) the Australian taxa are dispersed across the phylogeny temporally; (2) the Australian continent becomes progressively more isolated from other southern continents; and (3) a strengthened ACC (indicated by the black arrows) provides a new dispersal vector to Australia despite the presence of a strengthening Antarctic Polar Front (APF). The bottom palaeomaps are based on reconstructions in Lawver & Gahagan [9]. Penguin silhouettes show overall trend for decreasing body size in penguin evolution: Top, archaic giant stem penguin taxa; middle medium-sized stem penguin taxa; bottom, smaller crown penguin taxa (silhouette credit: Fir0002/Flagstaffotos (original photo), John E. McCormack, Michael G. Harvey, Brant C. Faircloth, Nicholas G. Crawford, Travis C. Glenn, Robb T. Brumfield & T. Michael Keesey, used under a CC BY 3.0 Attribution Unported Licence (http://creativecommons.org/licenses/by/3.0/))). Palaeoceanographic reconstructions after [9,72–74]. Palaeoceanographic abbreviations: EAC = East Australian Current, pEAC = palaeo-East Australian Current, pRG = palaeo-Ross Sea Gyre/Tasman Current, RG = Ross Sea Gyre. The relative strength of the ACC and APF is shown by thickening arrows and lines though time. Black arrow = cold currents, red arrows = warm currents.

The penguin fauna that we are familiar with today is markedly different to that which lived at the close of the Paleogene. Archaic giant penguins were present on all of the southern continents e.g. [2,3,10,55]. Yet no Miocene fossils of giant penguins have been found anywhere in the world apart from Australia. *Anthropodyptes gilli* is a giant penguin that existed in Australia 3–7 Ma after any other giant taxon went extinct. Based on its phylogenetic position (Fig 5) this could be interpreted as either: 1) giant body size was the plesiomorphic condition shared with other giant penguin taxa, or 2) *Anthropodyptes* evolved its giant size independently from small-bodied ancestors. Both scenarios are equally parsimonious and either interpretation could perhaps be a result of the isolation of the Australian continent.

Molecular data estimates that the age of the most recent common ancestor of living penguins is 20.4 Ma with most of the major extant groups diverging 11–16 Ma [45]. Previously, the oldest known crown genus for which there is fossil material has been *Spheniscus* (*S*. *muizoni*), which was originally dated as 11–13 Ma [16]. However more recent work has revised this age to 9.0–9.2 Ma [56]. This now means that the oldest reported crown group penguin fossils are a humerus and a radius dated to 10.2 Ma and assigned to *Spheniscus* sp. from East Antarctica [18] and *Madrynornis mirandus* from the Puerto Madryn Formation of Argentina (10.0 ± 0.3 Ma)[5].

The middle Miocene is thought to be the time when most crown clade penguin genera diverged [45]. By the late Miocene most stem taxa were replaced by crown clade taxa including extant genera. Stem Sphenisciformes are known to have survived until the Pliocene in Africa (i.e. *Dege hendeyi*) [39,57] and the middle Miocene in South America [41]. There are at least two stem taxa in the late Miocene of Australia: *Pseudaptenodytes macraei* and NMV P221273. In contrast to the other Southern Ocean landmasses, Australia possesses a late Neogene penguin fauna consisting solely of stem taxa, based on available evidence.

Despite not being included in the phylogenetic analysis, *Tasidyptes hunteri* merits discussion here. As noted above we recommend that the hypodigm of *Tasidyptes* should be restricted to the holotype synsacrum only. As the paratype tarsometatarsus and referred coracoid are most likely *Eudyptes* [36], it would appear that there are at least two taxa represented in the Hunter Island midden that are not *Eudyptula*. This may suggest the establishment in Australia of two other crown species in addition to *E*. *minor*; or perhaps the preservation of two separate vagrant dispersals by those taxa, a plausible scenario given that that several species of crown penguin are indeed occasional vagrants to Australia [58]. Irrespective of the problematic taxonomic status of *Tasidyptes*, the material raises the scenario that more than one species of crown penguin were resident in southeast Australia during the late Holocene. It also hints at an alternative route for the arrival of crown penguins to Australia via New Zealand and the Sub-Antarctic Islands. However, except for *Tasidyptes* and the original referred material, there is currently no evidence to support the dispersal of any penguin taxa via this route. Given that the *Tasidyptes* material is dated to 760 ± 70 ybp [16] and *Eudyptula minor* is estimated to have reached Australia 2.4 Ma [59] we consider it more likely (based on current evidence) that the initial dispersal of crown penguins to Australia arrived as a result of the strengthened ACC.

Nevertheless the lack of evidence for crown clade penguins in Australia until relatively recently is puzzling as fossil species of both *Aptenodytes* and *Pygoscelis* have been reported from New Zealand [60] (but see Ksepka and Clarke [39]), providing a minimum age estimate of middle Miocene–Pliocene for the presence of the crown group in the Australasian region. The uncertain age estimate for these fossils is explained by their discovery in eroded concretions found as float and lacking index fossils [60,61]. Stem Sphenisciformes continued to live in Australia long after the divergence of the crown clade and there is no evidence of crown Sphenisciformes in Australia by 6.0–7.5 Ma.

### The unique physical history of the Australian continent and its penguin fauna

Recent studies on the fossil penguin faunas of Antarctica, New Zealand, South Africa and South America [2–7,12,17,19,38,51,62–64] have converged on several key events in penguin history: the widespread dispersal of an archaic grade of giant penguins during the late Eocene; this grade going extinct by the Oligocene-Miocene boundary, replaced by more crownward smaller bodied forms in the early Miocene; the first occurrence of crown group penguins in the middle Miocene; and the dispersal of the crown clade to all the southern continents, by the end of the Miocene, hence becoming the dominant penguin group. Available evidence suggests that the post-Eocene history of penguins in Australia followed a different course to that of elsewhere, namely: giant penguins existed in the early Miocene; and multiple stem taxa occurred in the late Miocene, with no evidence of the crown clade. The disparate Cenozoic history of the Australian continent itself may have contributed to the unique evolutionary history of its penguins. Salient here are: (1) the final separation of Australia from Antarctica; (2) the northwards movement of Australia; and (3) the initiation and subsequent strengthening of the Antarctic Circumpolar Current (ACC).

#### Final separation of Australia from Antarctica

In the late Eocene Australia and the other southern continents were in much closer proximity than at present, being in the final stages of separation. Southern Australia was at ~60°S (~20° south of its current latitude) [9] and by 38 Ma, a 600–1000 km wide gulf (Australo-Antarctic Gulf) had developed between Australia and Antarctica (Fig 5), with depths of 4500 m in places [65]. It may be hypothesised that as a result of this proximity between the southern continents; there would be a close relationship between penguin taxa living in the region at this time. However, the poor resolution of this part of the phylogeny relative to other studies [7,39],prevents illumination of the relationships of the Australian taxa and therefore evaluation of biogeographic hypotheses.

#### The northward movement of Australia

Once Australia completed its separation from Antarctica ~34 Ma [9], it began moving steadily northwards. The growing distance between Australia and Antarctica, coupled with the opening of the Drake Passage and associated initiation of the ACC during the late Oligocene or even early Miocene [66–68]; plus establishment of a weak Antarctic Polar Front ~ 20 Ma (APF: Fig 5) [30,69,70], would have been a barrier to pelagic animal dispersal (Fig 5) [71] decreasing the likelihood of penguins dispersing to Australia from Antarctica (discussed below).

By 17.6–21.0 Ma, when *Anthropodyptes gilli* lived in Australia, this geographic isolation had become even more pronounced due to a well-developed APF. The northward drift of the Australian plate accelerated, leaving the polar region and entering temperate latitudes, with the southern continental margin then being situated at 45°–48°S [9]. The widening Australian-Antarctic seaway caused the developing ACC to shift northward, making it progressively more difficult for Antarctic taxa to disperse across the strengthening APF to Australia due to the longer distance they would have had to travel to escape entrainment in the ACC. Australia’s isolation from other Southern Ocean landmasses is further demonstrated by the decreasing fraction of austral taxa in the marine invertebrate fauna as Australia drew closer to Southeast Asia [72,73].

#### The initiation and subsequent strengthening of the Antarctic Circumpolar Current

The ACC began to flow during the Oligocene or early Miocene [9,66–68] as a result of the opening of the Drake Passage. At this early stage however, the ACC was still relatively weak, perhaps limiting its significance as a dispersal vector to Australia from the west-southwest. By the late Miocene, during which NMV P221273 and *Pseudaptenodytes macraei* occurred in Australia, the ACC and APF had strengthened, as a result of the formation of the deep water pathway around Antarctica and the collision of the Australia/New Guinea block with southeast Asia [69], which stemmed equatorial circulation from the Pacific into the Indian Ocean and forced the water southward, increasing the driving force of the ACC [9]. The strengthening of the ACC may have enhanced the role of this current as a dispersal vector for penguins north of the APF to reach Australia from the west/southwest. Using this stronger current, taxa from regions such as South America and South Africa may have dispersed eastward across the Indian and Southern oceans towards Australia. The large distances involved probably kept dispersal events to a minimum but the stronger vector current may have increased the likelihood of penguins successfully reaching Australia from other landmasses fringing the Southern Ocean north of the APF. Alternatively, penguin taxa may have dispersed to Australia from New Zealand throughout the Cenozoic, a much shorter distance than from Australia to Antarctica, South America or South Africa. Nevertheless, the polytomies resulting from our phylogenetic analysis prevent objective discrimination of more likely dispersal routes. Yet they do corroborate a change from the vicariance events seen previously in the Palaeogene to predominantly dispersal events proposed above during the Neogene with *Eudyptula minor* in a clade with taxa from South Africa and South America.

NMV P221273 and *P*. *macraei* resolve with taxa from New Zealand and South America in a polytomy one node below crown Sphenisciformes. Interestingly, the non-Australian taxa in this polytomy are all older than NMV P221273 and *P*. *macraei*, with only *Marplesornis* potentially being contemporaneous with the Australian taxa. However all taxa in this polytomy had to have diverged prior to 10.2 Ma (the age of the oldest known crown clade penguin), suggesting that Australian lineages may have been resident in Australia for a long time prior to their oldest fossil, potentially even before the strengthening of the ACC. NMV P221273 and *P*. *macraei* are amongst the last known stem taxa. Beyond Australia, crown group penguins had usurped earlier-diverging stem penguins by the late Miocene [38], or were at least living alongside the few remaining stem group taxa as in South Africa [36]. However, in the late Miocene of Australia, only stem taxa have been recorded thus far. Geographic isolation of Australia may have resulted in the survival of archaic stem penguins, until the potentially post-Miocene arrival of crown penguins. These concepts of the history of penguins in Australia are preliminary, and necessarily so due to their currently limited fossil record on this continent. Nonetheless, the phylogenetic and biogeographic hypotheses presented here represent a testable framework in which future fossil penguin discoveries in Australia may be interpreted.

## Supporting Information

S1 File Park et al 2015 matrix. (TXT)
